# Clinical analgesic efficacy of pectoral nerve block in patients undergoing breast cancer surgery: A systematic review and meta-analysis

**DOI:** 10.1097/MD.0000000000019614

**Published:** 2020-04-03

**Authors:** Qianchuang Sun, Shuyan Liu, Huiying Wu, Wenyue Kang, Shanshan Dong, Yunfeng Cui, Zhenxiang Pan, Kexiang Liu

**Affiliations:** a Department of Anesthesiology; b Department of Ultrasonic Diagnosis, The Second Hospital of Jilin University, Changchun; c Department of Anesthesiology, Hainan Provincial People's Hospital, Hainan; d Department of Cardiovascular Surgery, The Second Hospital of Jilin University, Changchun, China.

**Keywords:** breast cancer, meta-analysis, opioid consumption, pain scores, pectoral nerve block

## Abstract

**Background:**

Breast cancer is the most commonly diagnosed cancer in women, and more than half of breast surgery patients experience severe acute postoperative pain. This meta-analysis is designed to examine the clinical analgesic efficacy of Pecs block in patients undergoing breast cancer surgery.

**Methods:**

An electronic literature search of the Library of PubMed, EMBASE, Cochrane Library, and Web of Science databases was conducted to collect randomized controlled trials (RCTs) from inception to November 2018. These RCTs compared the effect of Pecs block in combination with general anesthesia (GA) to GA alone in mastectomy surgery. Pain scores, intraoperative and postoperative opioid consumption, time to first request for analgesia, and incidence of postoperative nausea and vomiting were analyzed.

**Results:**

Thirteen RCTs with 940 patients were included in our analysis. The use of Pecs block significantly reduced pain scores in the postanesthesia care unit (weighted mean difference [WMD] = −1.90; 95% confidence interval [CI], −2.90 to −0.91; *P* < .001) and at 24 hours after surgery (WMD = −1.01; 95% CI, −1.64 to −0.38; *P* < .001). Moreover, Pecs block decreased postoperative opioid consumption in the postanesthesia care unit (WMD = −1.93; 95% CI, −3.51 to −0.34; *P* = .017) and at 24 hours (WMD = −11.88; 95% CI, −15.50 to −8.26; *P* < .001). Pecs block also reduced intraoperative opioid consumption (WMD = −85.52; 95% CI, −121.47 to −49.56; *P* < .001) and prolonged the time to first analgesic request (WMD = 296.69; 95% CI, 139.91–453.48; *P* < .001). There were no statistically significant differences in postoperative nausea and vomiting and block-related complications.

**Conclusions:**

Adding Pecs block to GA procedure results in lower pain scores, less opioid consumption and longer time to first analgesic request in patients undergoing breast cancer surgery compared to GA procedure alone.

## Abstract 2

Supplemental Digital Content is available in the text

## 1 Introduction

Breast cancer is the most commonly diagnosed cancer in women.^[1]^ In 2018, it is estimated that there were 266,120 new cases of invasive breast cancer diagnosed in women in the United States, and more than 40,920 people will die from breast cancer.^[1,2]^ Most of the time, surgery is considered the primary treatment for breast cancer, while radiation therapy, chemotherapy, and hormone therapy are given as adjuvant therapies.^[3,4]^

More than half of breast surgery patients experience severe acute postoperative pain, and acute postoperative pain is followed by persistent pain in approximately 25% to 50% of patients.^[5,6]^ Severe acute pain is a risk factor for chronic pain following breast cancer surgery, which is associated with impaired quality of life.^[7,8]^ Regional anesthesia techniques can provide better acute pain control and improve patient satisfaction.^[9–11]^ The pectoral nerve (Pecs) block, a novel technique described by Blanco in 2011, can provide analgesia for breast surgery.^[12]^ In this new technique, local anesthetic is injected into the interfascial plane between the pectoralis major and minor muscles (Pecs I block) to anesthetize the medial and lateral pectoral nerves. Blanco and colleagues proposed a second version of the Pecs block in 2012, called “modified Pecs block” or Pecs block type II (Pecs II block).^[13]^ For Pecs II block, local anesthetic is deposited deeper to the Pecs I injection site and above the serratus anterior muscle at the third rib, which aims to block the pectoral nerves, the intercostobrachial, lateral branches of intercostal nerves III, IV, V, VI, and the long thoracic nerve.^[13]^ To date, a number of studies have confirmed that Pecs block is a simple and easy-to-learn technique that produces good analgesia for radical breast surgery^[14–17]^. However, some well-designed randomized controlled trials (RCTs) have failed to show that Pecs block can offer superior analgesia after breast surgery.^[18,19]^

The Pecs block is widely used for postoperative analgesia after breast surgery.^[13]^ Compared to thoracic paravertebral and thoracic epidural blocks, the Pecs block has less technical complexity and fewer complications.^[13,15]^ Is there enough evidence to support the use of Pecs block for radical mastectomy? In this study, we conducted a meta-analysis to evaluate clinical analgesic efficacy of Pecs block in patients undergoing breast cancer surgery.

## 2 Methods

Studies were performed in accordance with the PRISMA protocol (Supplementary Table S1).^[20]^

### 2.1 Study search strategy

We systematically searched the PubMed, EMBASE, Cochrane Library, and Web of Science databases from inception to November 2018. Medical subject headings and text words “pectoral nerve block, Pecs block, Pecs I and Pecs II blocks or PECS” and “breast cancer or radical mastectomy” were used to search for trials of interest. Details of the search strategies are summarized in Supplementary Table S2. The search was restricted to articles in the English language. In order to avoid omitting relevant clinical trials, we also searched conference summaries and references for potential eligible reports.

### 2.2 Selection criteria

Inclusion criteria were as follows:

(1) studies designed as RCTs;
(2) female patients undergoing breast cancer surgery;
(3) experimental groups treated with general anesthesia (GA) plus Pecs block, and the control group with GA alone;
(4) outcomes such as pain scores, postoperative opioid consumption (in the postanesthesia care unit [PACU] and at 24 hours after surgery), intraoperative fentanyl consumption, time to first request for analgesia, and incidence of postoperative nausea and vomiting (PONV).

Exclusion criteria were as follows:

(1) non-RCTs;
(2) reviews, letters, abstracts, editorials, or studies reporting insufficient data;
(3) no control group.

### 2.3 Data extraction

Two reviewers (QCS, SYL) independently extracted data from the selected studies. Disagreements were resolved by group consensus. The following information was extracted from studies that met the inclusion criteria: first author, year of publication, country, number of patients, study design, and outcome measures. If data were presented as median and interquartile range, we contacted the author for necessary data. Failing that, the mean was considered to be equivalent to the median, and the standard deviation = interquartile range/1.35.^[21]^

### 2.4 Outcomes

Pain scores (in PACU and at 1, 2, 3, 6, 12, and 24 hours after surgery) were defined as primary outcome measures. Pain scores were presented as a visual analog scale (VAS) (0 = no pain and 10 = worst possible pain). Secondary outcomes were postoperative opioid consumption (in the PACU and at 24 hours after surgery), intraoperative fentanyl consumption, time to first request for analgesia, incidence of PONV, and block-related complications. Opioid consumption was converted to morphine equivalent doses, where intravenous (i.v.) morphine 10 mg = i.v. sufentanil 10 μg = i.v. tramadol 100 mg = i.v. fentanyl 0.1 mg = i.v. remifentanil 0.05 mg.^[22–26]^

### 2.5 Quality assessment

We used the Cochrane Risk of Bias Tool to assess the quality of the included studies.^[27]^ The evaluation should include the following domains:

(1) random sequence generation;
(2) allocation concealment;
(3) blinding of participants and personnel;
(4) blinding of outcome assessment;
(5) incomplete outcome data;
(6) selective reporting;
(7) other bias.

Each of these domains was judged as low risk, high risk, or unclear risk. Any disagreements were resolved by discussion.

For the assessment of publication bias of the studies included in the final analysis, both Begg rank correlation and Egger linear regression tests were performed.^[28,29]^

### 2.6 Statistical analysis

All statistical analyses were performed in Stata 14.0 (Stata Corp, College Station, TX) and Review Manager 5.3 (The Nordic Cochrane Centre, The Cochrane Collaboration, Copenhagen, 2014). Risk ratios with 95% confidence intervals (CIs) were calculated for dichotomous data, and weighted mean differences with 95% CIs were calculated for continuous variables. Heterogeneity was measured by *I*^2^, with *I*^2^ > 50% indicating significant heterogeneity. If *I*^2^ ≤50%, the fixed effects model was used; if *I*^2^ > 50%, a random effects model was used, and the heterogeneity was assessed. Subgroup analyses were performed for the outcome measures, according to time of block (before GA, after GA, or after surgery) and local anesthetic types (ropivacaine, bupivacaine, or levobupivacaine). Sensitivity analyses were performed by excluding 1 study each time to evaluate the influence of a single study on the overall estimate.^[30]^ This is a meta-analysis. Thus, ethical approval was not necessary and the informed consent was not given.

## 3 Results

### 3.1 Literature search

**Figure 1:**
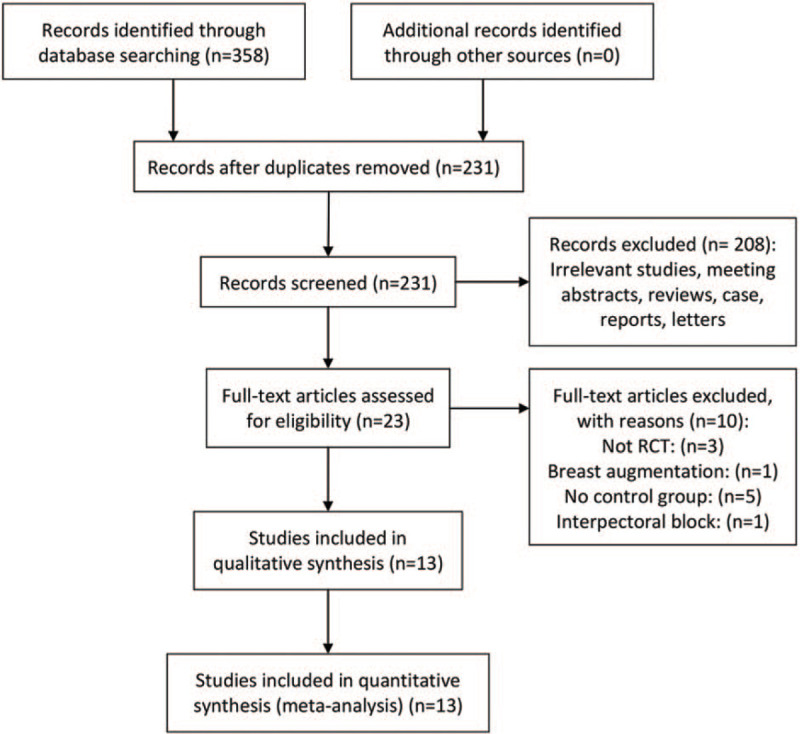
Flowchart of the study selection.

Figure 1 presents a summary of the study search process. A total of 358 relevant studies were initially identified. Of these, 127 were excluded due to duplication. After screening of the titles and abstracts, 208 were further excluded. By reading the full text of the remaining 23 articles, 10 of them were additionally excluded because they failed to meet the inclusion criteria. Thus, 13 RCTs with 940 patients were finally assessed in this meta-analysis.^[14–19,31–37]^

### 3.2 Study characteristics

**Figure 2:**
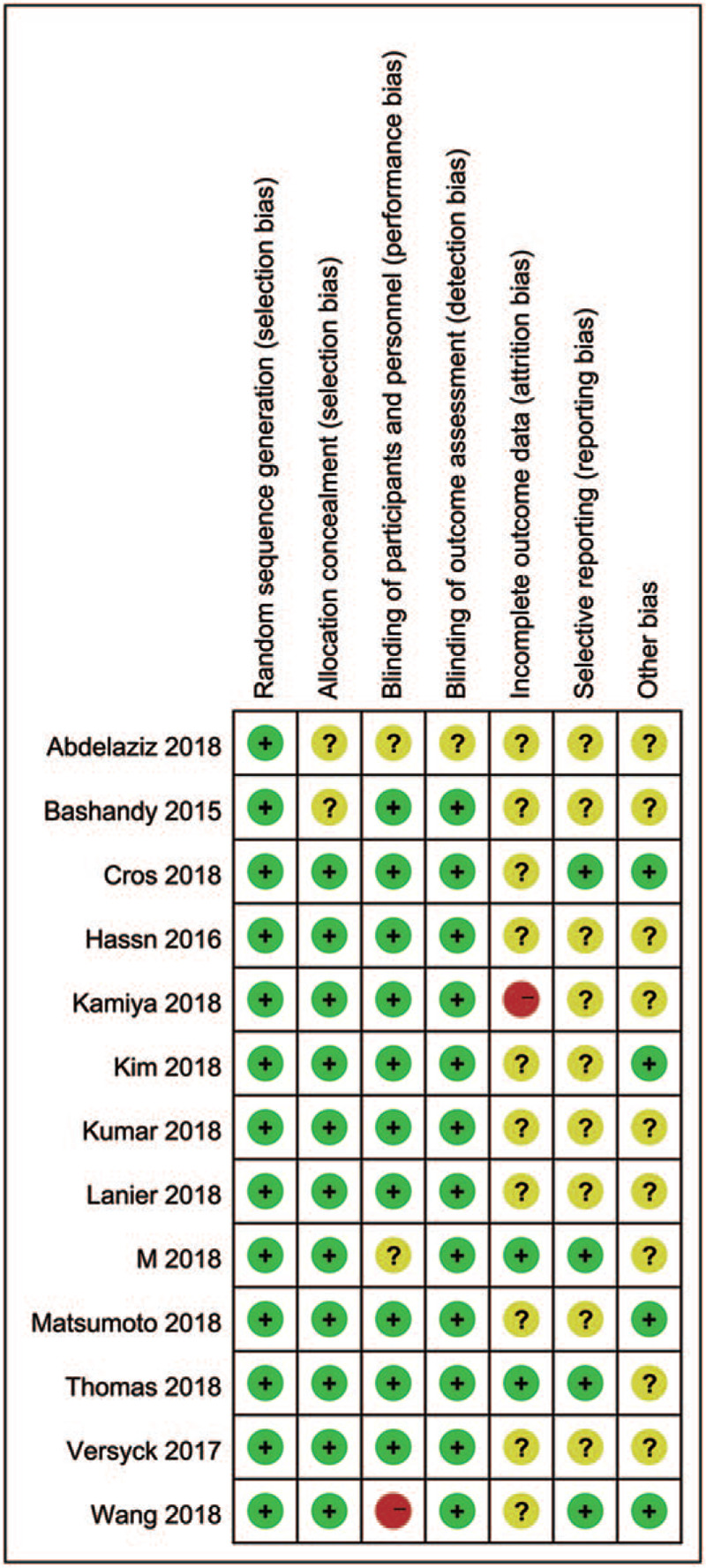
Risk of bias summary.

The characteristics of the included studies are summarized in Table 1. Thirteen trials compared Pecs block in combination with GA to GA alone in mastectomy surgery. Of these 13 trials, 8 performed Pecs block after the induction of anesthesia, 2 performed before the induction of anesthesia, and 2 applied at the completion of the surgery. Eleven studies underwent ultrasound guided Pecs block and the other 2 applied Pecs block under direct visualization. Among these 13 trials, 5 used ropivacaine, 6 used bupivacaine, and 2 others used levobupivacaine. Pain scores were reported in all included trials. The risk assessment of the included studies is presented in Figure 2. Eleven trials did not have a high risk of bias for any of the evaluated criteria. One study had a high risk of detection bias, while 1 study had a high risk of attrition bias.

**Table 1:**
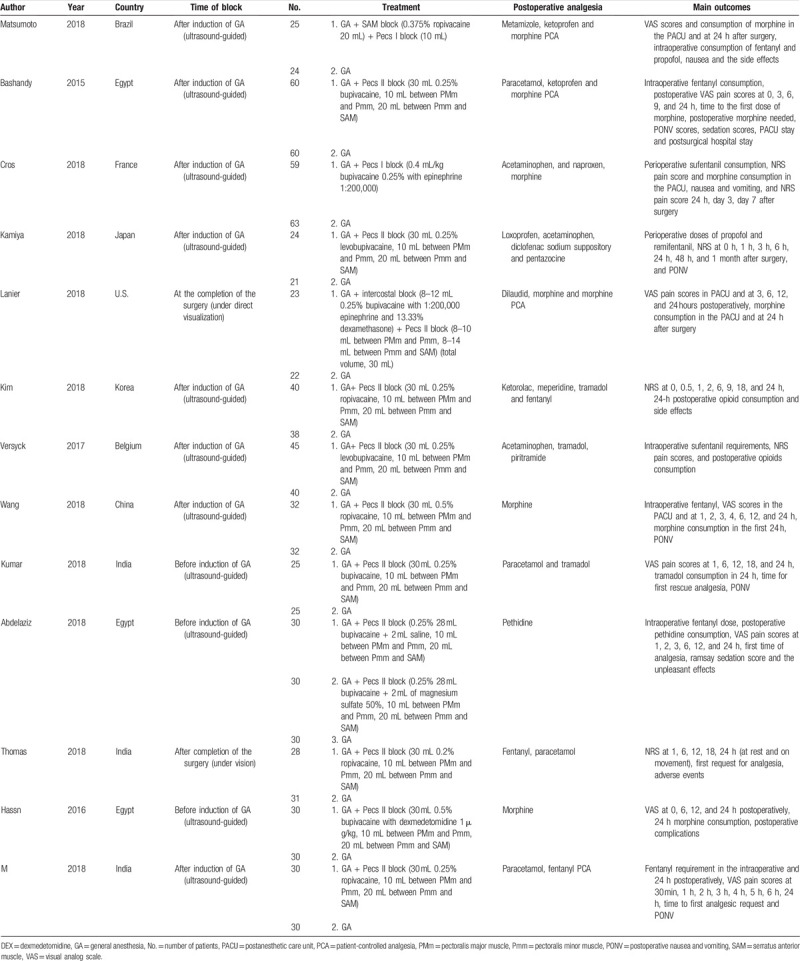
Trial characteristics.

### 3.3 Postoperative pain scores

**Figure 3:**
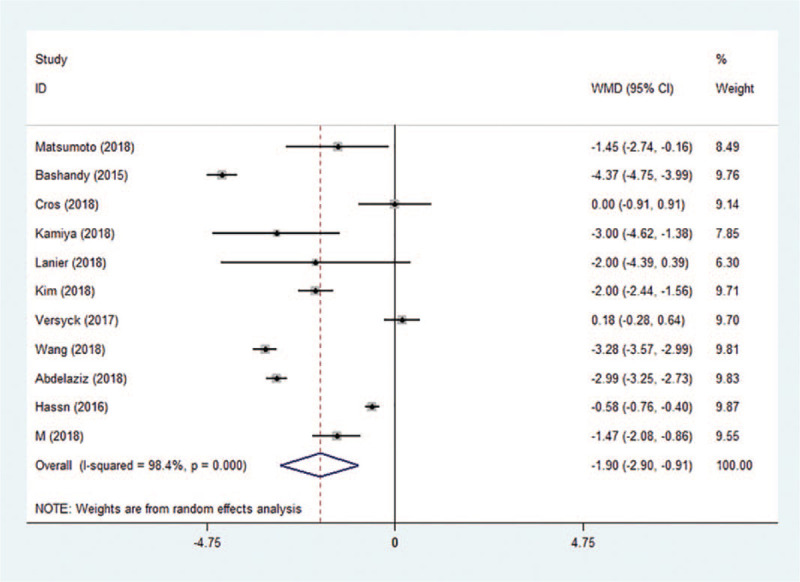
Forest plot of pain scores in the PACU. CI = confidence interval, PACU = pain scores in the postanesthesia care unit, WMD = weighted mean difference.

**Figure 4:**
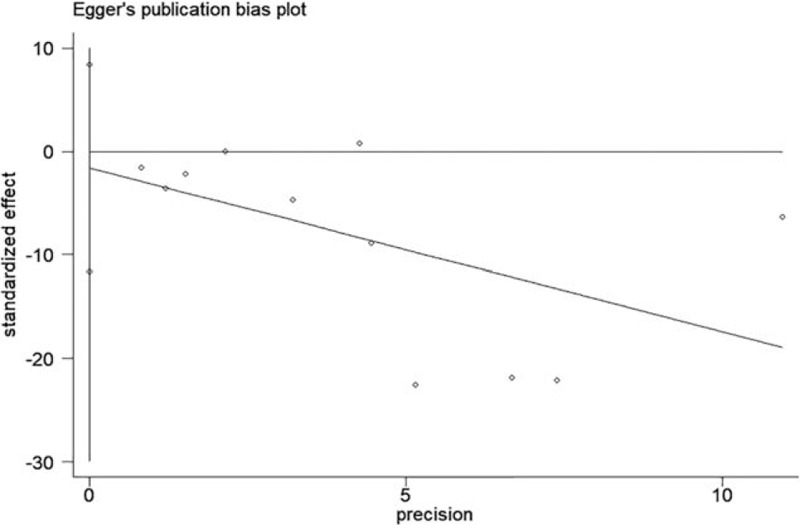
Begg funnel plot of pain scores in the PACU. PACU = pain scores in the postanesthesia care unit, WMD = weighted mean difference.

**Figure 5:**
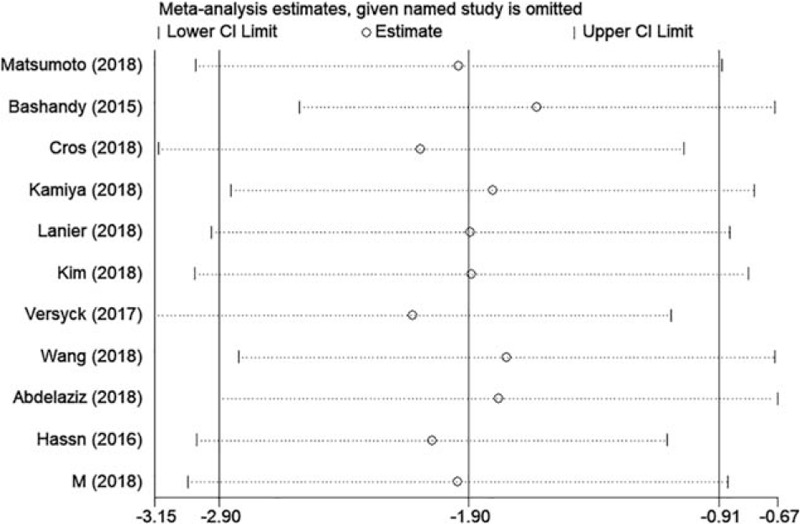
Sensitivity analysis of pain scores in the PACU. CI = confidence interval, PACU = pain scores in the postanesthesia care unit.

Pain scores were reported in all included trials. Pain scores (in the PACU and at 1, 2, 3, 6, 12, and 24 hours after surgery) are summarized in Table 2. At all time points, significantly lower pain scores were reported by patients receiving Pecs block compared to the control group. Pain scores decreased from −1.90 (95% CI: −2.90 to −0.91, *P* < .001, *I*^2^ = 98.4%) in the PACU (Fig. 3) to −1.01 (95% CI: −1.64 to −0.38, *P* < .001, *I*^2^ = 97.1%) at 24 hours postoperatively. No evidence of publication bias was observed on Begg funnel plot (*P* = 1.000, Fig. 4) or Egger test (*P* = .727). Sensitivity analysis did not significantly alter the summarized results (Fig. 5).

**Table 2:**
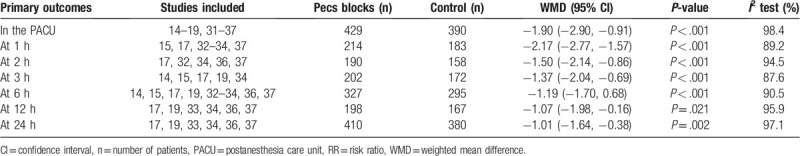
Pain scores postoperatively.

### 3.4 Postoperative opioid consumption

**Figure 6:**
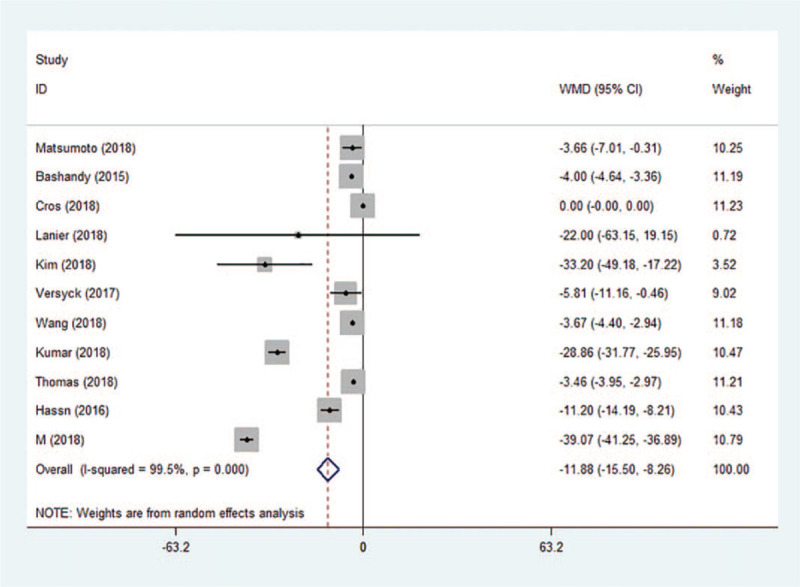
Forest plot of morphine equivalents 24 h postoperatively. CI = confidence interval, WMD = weighted mean difference.

Postoperative opioid consumption (converted to IV morphine equivalents) was assessed in the PACU in 4 trials and at 24 hours in 13 trials. The use of Pecs block reduced opioid consumption by an average of −1.93 mg (95% CI: −3.51 to −0.34, *P* = .017, *I*^2^ = 17.2%) in the PACU (S.1) and −11.88 mg (95% CI: −15.50 to −8.26, *P* < .001, *I*^2^ = 99.5%) at 24 hours (Fig. 6). Although Egger test indicated publication bias (*P* = .010), Begg test was not significant (*P* = .161). Sensitivity analysis did not significantly alter the summarized results.

### 3.5 Intraoperative opioid consumption

**Figure 7:**
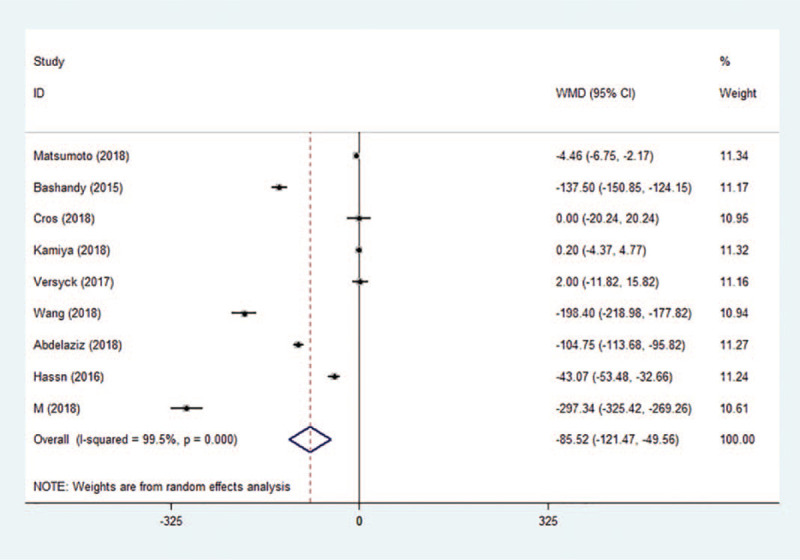
Forest plot of intraoperative fentanyl equivalents. CI = confidence interval, WMD = weighted mean difference.

Nine of the 13 studies measured the intraoperative opioid consumption (converted to IV fentanyl equivalents). Compared to the control group, Pecs block was effective in reducing intraoperative opioid consumption by −85.52 μg (95% CI: −121.47 to −49.56, *P* < .001, *I*^2^ = 99.5%) (Fig. 7). Although Egger test indicated publication bias (*P* = .022), Begg test was not significant (*P* = .175). Sensitivity analysis did not significantly alter the summarized results.

### 3.6 First request for analgesia

**Figure 8:**
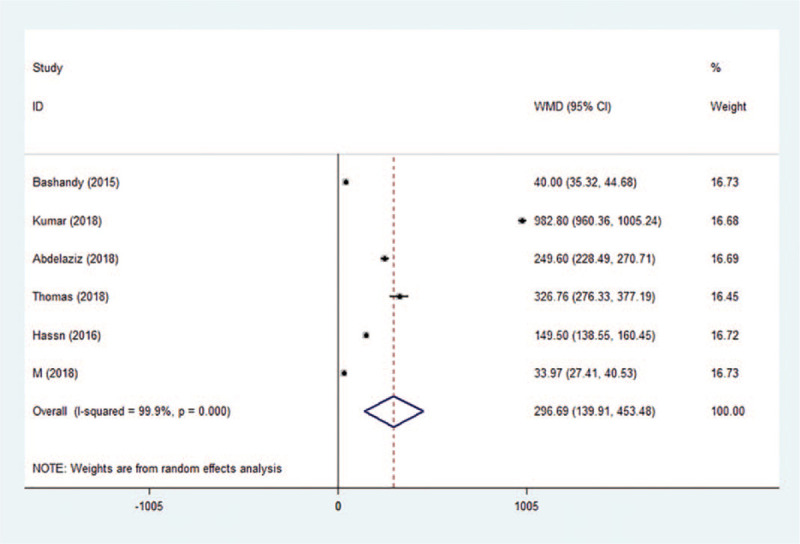
Forest plot of time to first request for analgesia. CI = confidence interval, WMD = weighted mean difference.

First requests for analgesia were available in 6 studies. On average, Pecs block delayed the time to first request for analgesia by 296.69 minutes (95% CI: 139.91–453.48, *P* < .001, *I*^2^ = 99.9%) (Fig. 8). No evidence of publication bias was observed on Begg test (*P* = .133) or Egger test (*P* = .109). Sensitivity analysis did not significantly alter the summarized results.

### 3.7 PONV and block-related complications

Five studies investigated the incidence of PONV. There was no statistically significant difference in PONV (S.2). One study reported block-related complications such as bleeding and hematoma in 3 patients. However, no block-related complications were reported in the other 12 studies.

### 3.8 Subgroup analyses

Subgroup analyses are shown in Table 3. Use of time of block (before/after induction of GA or after completion of the surgery) and local anesthetic types (ropivacaine, bupivacaine, or levobupivacaine) may account for heterogeneity in some of the findings.

**Table 3:**
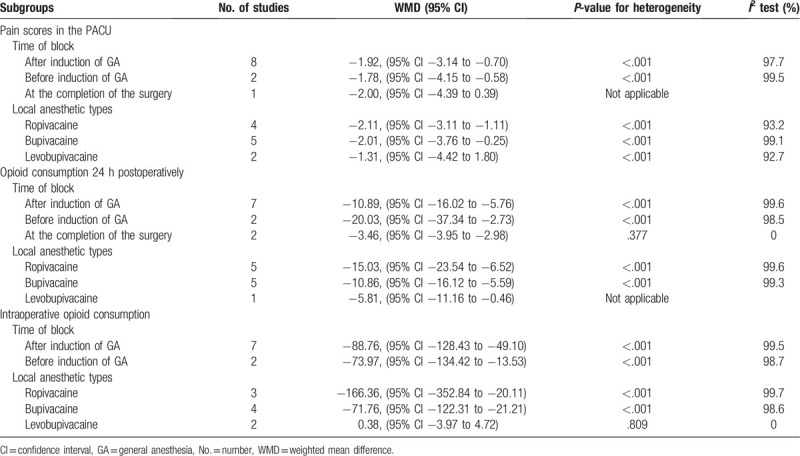
Subgroup analyses.

## 4 Discussion

This is one of the first meta-analyses to examine the clinical analgesic efficacy of Pecs block in patients undergoing breast cancer surgery. Our meta-analysis showed that the use of Pecs block significantly reduced VAS pain scores up to 24 hours postoperatively. In addition, breast cancer patients receiving Pecs block had significantly less intraoperative and postoperative opioid consumption than the control group. The analgesic effect of Pecs block was also demonstrated by a longer time to the first request for analgesia. There was no statistically significant difference in PONV and complications related to Pecs block.

Surgery is the first choice of treatment for breast cancer, and regional anesthesia may potentially reduce the post-mastectomy pain syndrome.^[38]^ It has been implicated that regional anesthesia could reduce tumor recurrence and metastases after mastectomy.^[3]^ Kairaluoma and Ibarra suggested that paravertebral block significantly reduces the acute and chronic pain compared to the sham block.^[39,40]^ As the thoracic paravertebral space is in close relation to the pleural space, thoracic paravertebral block has potential risks of pneumothorax and total spinal anesthesia.^[41]^ In recent years, a less invasive and more effective Pecs block has become popular for perioperative pain control in patients undergoing breast cancer surgery.^[12–15]^ In this meta-analysis, the use of Pecs block significantly decreased VAS pain scores by 1.90 points in the PACU and 2.17 points at postoperative 1 hour. Although the reduction of VAS pain scores reduced to 1.01 points at 24 postoperatively, the difference remained significant. Moreover, Pecs block prolonged the time to first analgesic request by 296.69 minutes. The lower VAS pain scores resulted in reduced chronic pain, better sleep, higher patient satisfaction, and less hospital readmission.^[36]^

Although conventional opioid analgesics remain the mainstay of postoperative pain management, their use may be limited by potentially harmful effects.^[42,43]^ Steyaert and colleagues demonstrated that patients who needed opioids in the immediate postoperative period were associated with the presence of chronic pain after mastectomy with axillary lymph node dissection.^[44]^ Therefore, a multimodal approach to improve postoperative analgesia must be utilized, including local infiltration, regional anesthesia, and nonopioid analgesics. ^[42,43]^ In the current meta-analysis, the use of Pecs block decreased intraoperative (fentanyl equivalent) opioid consumption by −85.52 μg. However, we found levobupivacaine failed to decrease intraoperative opioid consumption after performing subgroup analysis. Only 2 studies involving 130 participants investigated the efficacy of levobupivacaine. Because of relative smaller sample size, the result should be interpreted with caution as the statistical power of this analysis is low. Furthermore, postoperative (morphine equivalent) opioid consumption was 1.93 mg lower in the PACU and 11.88 mg lower at 24 hours. The opioid sparing effect led to increased patient satisfaction and decreased length of hospital stay.^[17,36]^

This meta-analysis has several limitations that should be considered. First, high heterogeneity was found in some outcome measures. Although subgroup analyses (time of block and local anesthetic types) and sensitivity analyses were performed to identify the potential heterogeneity, we failed to change the heterogeneity. Second, despite a comprehensive search strategy and lack of language restriction, we found publication bias in the analysis of intraoperative and postoperative opioid consumption when we performed Egger test. However, this was not confirmed with Begg test, which is less susceptible to false positive results. ^[45]^ Third, 10 included studies performed Pecs block after induction when patients were unconscious. The quality of the block was not assessed before surgery, which might contribute to the heterogeneity of the analysis. Fourth, although opioid doses were converted to fentanyl and morphine equivalent doses, the calculations might result in some degree of variation. Lastly, due to insufficient information from original trials, we could not evaluate the efficacy of Pecs block on important outcomes such as sensory block duration, length of hospital stay, postoperative chronic breast pain, and tumor recurrence and metastases.

In conclusion, our meta-analysis indicated that adding Pecs block to GA procedures led to lower VAS pain scores, more significant opioid sparing, and longer time to first analgesic request in patients undergoing breast cancer surgery compared with GA procedures alone. Further studies are needed to investigate the long-term outcomes such as postoperative chronic pain, tumor recurrence and metastases, and recovery of shoulder function in these patients.

## Author contributions

**Conceptualization:** Qianchuang Sun, Zhenxiang Pan, Kexiang Liu.

**Data curation:** Shuyan Liu.

**Formal analysis:** Shuyan Liu, Huiying Wu, Wenyue Kang, Shanshan Dong, Yunfeng Cui.

**Methodology:** Shuyan Liu.

**Resources:** Wenyue Kang.

**Writing – original draft:** Qianchuang Sun.

**Writing – review & editing:** Zhenxiang Pan, Kexiang Liu.

## Supplementary Material

Supplemental Digital Content

## Supplementary Material

Supplemental Digital Content

## Supplementary Material

Supplemental Digital Content

## Supplementary Material

Supplemental Digital Content

## References

[1] Smith RA Andrews KS Brooks D Cancer screening in the United States, 2018: a review of current American Cancer Society guidelines and current issues in cancer screening. CA Cancer J Clin 2018;68:297–316. 29846940 10.3322/caac.21446

[2] Siegel RL Miller KD Jemal A Cancer statistics, 2018. CA Cancer J Clin 2018;68:7–30. 29313949 10.3322/caac.21442

[3] Exadaktylos AK Buggy DJ Moriarty DC Can anesthetic technique for primary breast cancer surgery affect recurrence or metastasis? Anesthesiology 2006;105:660–4. 17006061 10.1097/00000542-200610000-00008 PMC1615712

[4] Early Breast Cancer Trialists’ Collaborative Group Effects of chemotherapy and hormonal therapy for early breast cancer on recurrence and 15-year survival: an overview of the randomised trials. Lancet 2005;365:1687–717. 15894097 10.1016/S0140-6736(05)66544-0

[5] Fecho K Miller NR Merritt SA Acute and persistent postoperative pain after breast surgery. Pain Med 2009;10:708–15. 19453965 10.1111/j.1526-4637.2009.00611.x

[6] Gartner R Jensen MB Nielsen J Prevalence of and factors associated with persistent pain following breast cancer surgery. JAMA 2009;302:1985–92. 19903919 10.1001/jama.2009.1568

[7] Poleshuck EL Katz J Andrus CH Risk factors for chronic pain following breast cancer surgery: a prospective study. J Pain 2006;7:626–34. 16942948 10.1016/j.jpain.2006.02.007 PMC6983301

[8] Kehlet H Jensen TS Woolf CJ Persistent postsurgical pain: risk factors and prevention. Lancet 2006;367:1618–25. 16698416 10.1016/S0140-6736(06)68700-X

[9] Abdallah FW Morgan PJ Cil T Ultrasound-guided multilevel paravertebral blocks and total intravenous anesthesia improve the quality of recovery after ambulatory breast tumor resection. Anesthesiology 2014;120:703–13. 24071616 10.1097/ALN.0000436117.52143.bc

[10] Ilfeld BM Madison SJ Suresh PJ Treatment of postmastectomy pain with ambulatory continuous paravertebral nerve blocks: a randomized, triple-masked, placebo-controlled study. Reg Anesth Pain Med 2014;39:89–96. 24448512 10.1097/AAP.0000000000000035 PMC3944096

[11] Wu CL Raja SN Treatment of acute postoperative pain. Lancet 2011;377:2215–25. 21704871 10.1016/S0140-6736(11)60245-6

[12] Blanco R The ’pecs block’: a novel technique for providing analgesia after breast surgery. Anaesthesia 2011;66:847–8. 10.1111/j.1365-2044.2011.06838.x 21831090

[13] Blanco R Fajardo M Parras Maldonado T Ultrasound description of Pecs II (modified Pecs I): a novel approach to breast surgery. Rev Esp Anestesiol Reanim 2012;59:470–5. 22939099 10.1016/j.redar.2012.07.003

[14] Bashandy GM Abbas DN Pectoral nerves I and II blocks in multimodal analgesia for breast cancer surgery: a randomized clinical trial. Reg Anesth Pain Med 2015;40:68–74. 25376971 10.1097/AAP.0000000000000163

[15] Kamiya Y Hasegawa M Yoshida T Impact of pectoral nerve block on postoperative pain and quality of recovery in patients undergoing breast cancer surgery: a randomised controlled trial. Eur J Anaesthesiol 2018;35:215–23. 29227351 10.1097/EJA.0000000000000762

[16] Versyck B van Geffen GJ Van Houwe P Prospective double blind randomized placebo-controlled clinical trial of the pectoral nerves (Pecs) block type II. J Clin Anesth 2017;40:46–50. 28625445 10.1016/j.jclinane.2017.03.054

[17] Wang K Zhang X Zhang T The efficacy of ultrasound-guided type II pectoral nerve blocks in perioperative pain management for immediate reconstruction after modified radical mastectomy: a prospective, randomized study. Clin J Pain 2018;34:231–6. 28654558 10.1097/AJP.0000000000000529

[18] Cros J Sengès P Kaprelian S Pectoral I block does not improve postoperative analgesia after breast cancer surgery: a randomized, double-blind, dual-centered controlled trial. Reg Anesth Pain Med 2018;43:596–604. 29672368 10.1097/AAP.0000000000000779

[19] Lanier ST Lewis KC Kendall MC Intraoperative nerve blocks fail to improve quality of recovery after tissue expander breast reconstruction: a prospective, double-blinded, randomized, placebo-controlled clinical trial. Plast Reconstr Surg 2018;141:590–7. 29481391 10.1097/PRS.0000000000004104

[20] Liberati A Altman DG Tetzlaff J The PRISMA statement for reporting systematic reviews and meta-analyses of studies that evaluate health care interventions: explanation and elaboration. PLoS Med 2009;6:e1000100. 19621070 10.1371/journal.pmed.1000100 PMC2707010

[21] Wan X Wang W Liu J Estimating the sample mean and standard deviation from the sample size, median, range and/or interquartile range. BMC Med Res Methodol 2014;14:135. 25524443 10.1186/1471-2288-14-135 PMC4383202

[22] Pereira J Lawlor P Vigano A Equianalgesic dose ratios for opioids. A critical review and proposals for long-term dosing. J Pain Symptom Manage 2001;22:672–87. 11495714 10.1016/s0885-3924(01)00294-9

[23] Knotkova H Fine PG Portenoy RK Opioid rotation: the science and the limitations of the equianalgesic dose table. J Pain Symptom Manage 2009;38:426–39. 19735903 10.1016/j.jpainsymman.2009.06.001

[24] Michelsen LG Salmenpera M Hug CC Jr Anesthetic potency of remifentanil in dogs. Anesthesiology 1996;84:865–72. 8638841 10.1097/00000542-199604000-00014

[25] Sun Q Liu S Wu H Dexmedetomidine as an adjuvant to local anesthetics in transversus abdominis plane block: a systematic review and meta-analysis. Clin J Pain 2018;35:375–84. 10.1097/AJP.0000000000000671 PMC6410974 30475260

[26] Santonocito C Noto A Crimi C Remifentanil-induced postoperative hyperalgesia: current perspectives on mechanisms and therapeutic strategies. Local Reg Anesth 2018;11:15–23. 29670398 10.2147/LRA.S143618 PMC5898588

[27] Higgins JP Altman DG Gotzsche PC The Cochrane Collaboration's tool for assessing risk of bias in randomised trials. BMJ 2011;343:d5928. 22008217 10.1136/bmj.d5928 PMC3196245

[28] Begg CB Mazumdar M Operating characteristics of a rank correlation test for publication bias. Biometrics 1994;50:1088–101. 7786990

[29] Egger M Davey Smith G Schneider M Bias in meta-analysis detected by a simple, graphical test. BMJ 1997;315:629–34. 9310563 10.1136/bmj.315.7109.629 PMC2127453

[30] Patsopoulos NA Evangelou E Ioannidis JP Sensitivity of between-study heterogeneity in meta-analysis: proposed metrics and empirical evaluation. Int J Epidemiol 2008;37:1148–57. 18424475 10.1093/ije/dyn065 PMC6281381

[31] Matsumoto M Flores EM Kimachi PP Benefits in radical mastectomy protocol: a randomized trial evaluating the use of regional anesthesia. Sci Rep 2018;8:7815. 29777144 10.1038/s41598-018-26273-z PMC5959858

[32] Kim DH Kim S Kim CS Efficacy of pectoral nerve block type II for breast-conserving surgery and sentinel lymph node biopsy: a prospective randomized controlled study. Pain Res Manag 2018;2018:4315931. 29861803 10.1155/2018/4315931 PMC5976903

[33] Kumar S Goel D Sharma SK A randomised controlled study of the post-operative analgesic efficacy of ultrasound-guided pectoral nerve block in the first 24 h after modified radical mastectomy. Indian J Anaesth 2018;62:436–42. 29962525 10.4103/ija.IJA_523_17 PMC6004763

[34] Abdelaziz Ahmed AA Efficacy of pectoral nerve block using bupivacaine with or without magnesium sulfate. Anesth Essays Res 2018;12:440–5. 29962613 10.4103/aer.AER_37_18 PMC6020587

[35] Thomas M Philip FA Mathew AP Intraoperative pectoral nerve block (Pec) for breast cancer surgery: a randomized controlled trial. J Anaesthesiol Clin Pharmacol 2018;34:318–23. 30386013 10.4103/joacp.JOACP_191_17 PMC6194828

[36] Hassn AMA Zanfaly HE Biomy TA Pre-emptive analgesia of ultrasound-guided pectoral nerve block II with dexmedetomidine–bupivacaine for controlling chronic pain after modified radical mastectomy. Res Opin Anesth Intens Care 2016;3:6.

[37] M N Pandey RK Sharma A Pectoral nerve blocks to improve analgesia after breast cancer surgery: a prospective, randomized and controlled trial. J Clin Anesth 2018;45:12–7. 29241077 10.1016/j.jclinane.2017.11.027

[38] Humble S Dalton A Li L A systematic review of therapeutic interventions to reduce acute and chronic post-surgical pain after amputation, thoracotomy or mastectomy. Eur J Pain 2015;19:451–65. 25088289 10.1002/ejp.567 PMC4405062

[39] Kairaluoma PM Bachmann MS Rosenberg PH Preincisional paravertebral block reduces the prevalence of chronic pain after breast surgery. Anesth Analg 2006;103:703–8. 16931684 10.1213/01.ane.0000230603.92574.4e

[40] Ibarra MM GC##SC Vicente GU Chronic postoperative pain after general anesthesia with or without a single-dose preincisional paravertebral nerve block in radical breast cancer surgery. Rev Esp Anestesiol Reanim 2011;58:290–4. 21692253 10.1016/s0034-9356(11)70064-0

[41] Albi-Feldzer A Duceau B Nguessom W A severe complication after ultrasound-guided thoracic paravertebral block for breast cancer surgery: total spinal anaesthesia: a case report. Eur J Anaesthesiol 2016;33:949–51. 27801748 10.1097/EJA.0000000000000536

[42] Gan TJ Poorly controlled postoperative pain: prevalence, consequences, and prevention. J Pain Res 2017;10:2287–98. 29026331 10.2147/JPR.S144066 PMC5626380

[43] Garimella V Cellini C Postoperative pain control. Clin Colon Rectal Surg 2013;26:191–6. 24436674 10.1055/s-0033-1351138 PMC3747287

[44] Steyaert A Forget P Dubois V Does the perioperative analgesic/anesthetic regimen influence the prevalence of long-term chronic pain after mastectomy? J Clin Anesth 2016;33:20–5. 27555127 10.1016/j.jclinane.2015.07.010

[45] Peters JL Sutton AJ Jones DR Comparison of two methods to detect publication bias in meta-analysis. JAMA 2006;295:676–80. 16467236 10.1001/jama.295.6.676

