# Supplementary material for: Clinical analgesic efficacy of pectoral nerve block in patients undergoing breast cancer surgery: A systematic review and meta-analysis
Source: Medicine (Baltimore). 2020 Apr 3;99(14):e19614. doi: 10.1097/MD.0000000000019614 (PMC7440076; doi:10.1097/MD.0000000000019614)
Supplement: Supplemental Digital Content [file medi-99-e19614-s002.doc]

**Supplementary Table S2. Search strategies**

| **Search** | **Query** |
| --- | --- |
| #1 | Nerve, Thoracic |
| #2 | Nerves, Thoracic |
| #3 | Thoracic Nerve |
| #4 | Pectoral Nerves |
| #5 | Nerve, Pectoral |
| #6 | Nerves, Pectoral |
| #7 | Pectoral Nerve |
| #8 | OR/#1-7 |
| #9 | Block |
| #10 | Breast Neoplasm |
| #11 | Neoplasm, Breast |
| #12 | Breast Tumors |
| #13 | Breast Tumor |
| #14 | Tumor, Breast |
| #15 | Tumors, Breast |
| #16 | Neoplasms, Breast |
| #17 | Breast Cancer |
| #18 | Cancer, Breast |
| #19 | Mammary Cancer |
| #20 | Cancer, Mammary |
| #21 | Cancers, Mammary |
| #22 | Mammary Cancers |
| #23 | Malignant Neoplasm of Breast |
| #24 | Breast Malignant Neoplasm |
| #25 | Breast Malignant Neoplasms |
| #26 | Malignant Tumor of Breast |
| #27 | Breast Malignant Tumor |
| #28 | Breast Malignant Tumors |
| #29 | Cancer of Breast |
| #30 | Cancer of the Breast |
| #31 | Mammary Carcinoma, Human |
| #32 | Carcinoma, Human Mammary |
| #33 | Carcinomas, Human Mammary |
| #34 | Human Mammary Carcinomas |
| #35 | Mammary Carcinomas, Human |
| #36 | Human Mammary Carcinoma |
| #37 | Mammary Neoplasms, Human |
| #38 | Human Mammary Neoplasm |
| #39 | Human Mammary Neoplasms |
| #40 | Neoplasm, Human Mammary |
| #41 | Neoplasms, Human Mammary |
| #42 | Mammary Neoplasm, Human |
| #43 | Breast Carcinoma |
| #44 | Breast Carcinomas |
| #45 | Carcinoma, Breast |
| #46 | Carcinomas, Breast |
| #47 | OR/#10-46 |
| #48 | # 8AND #9 AND #47 |
