# Supplementary figures and images for: Clinical analgesic efficacy of pectoral nerve block in patients undergoing breast cancer surgery: A systematic review and meta-analysis
Source: Medicine (Baltimore). 2020 Apr 3;99(14):e19614. doi: 10.1097/MD.0000000000019614 (PMC7440076; doi:10.1097/MD.0000000000019614)

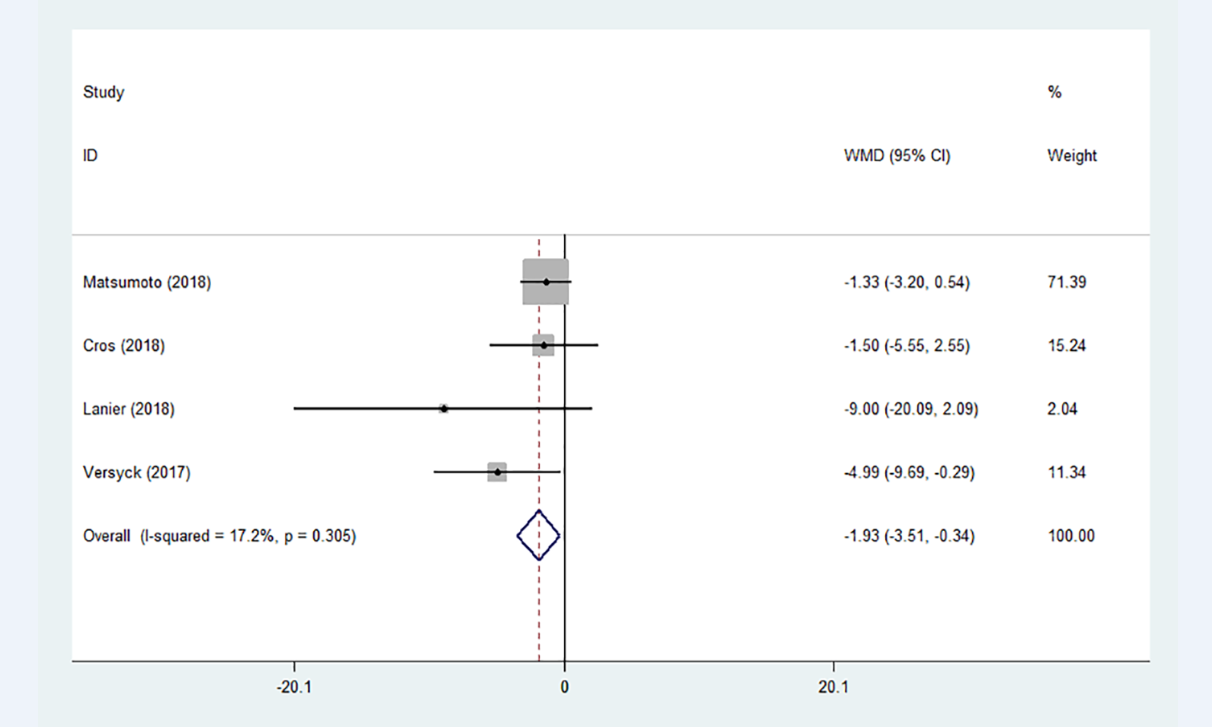

Supplement: Supplemental Digital Content [file medi-99-e19614-s003.doc]

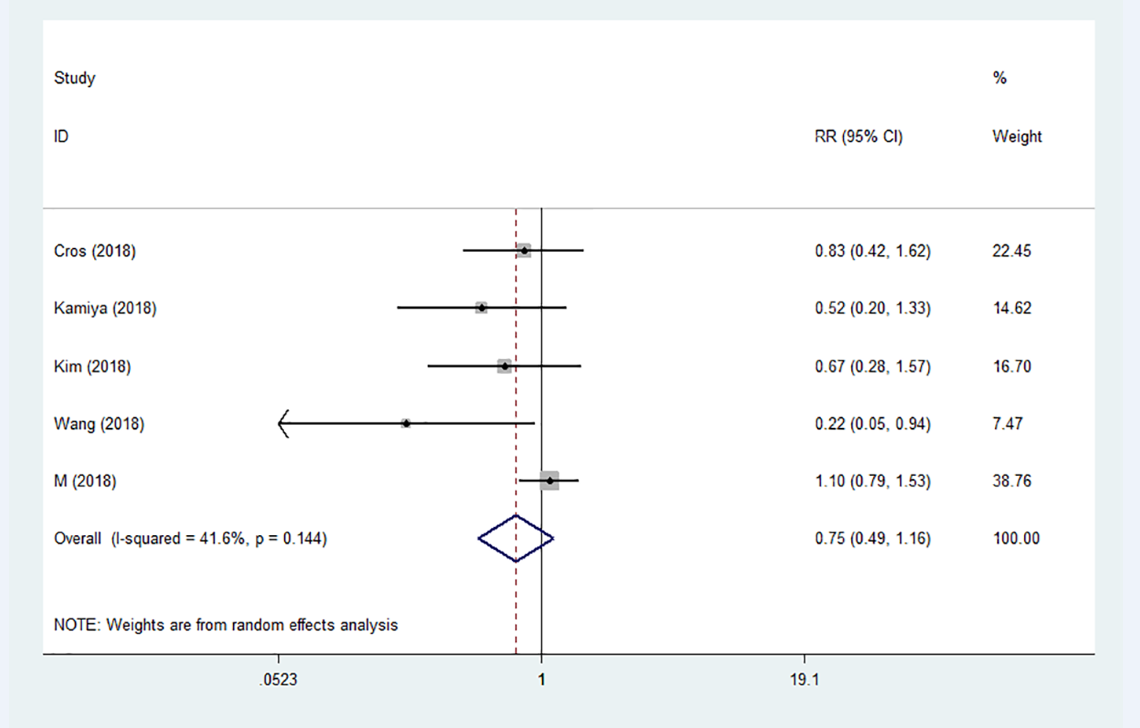

Supplement: Supplemental Digital Content [file medi-99-e19614-s004.doc]
